# Breakthrough cases of Omicron and Delta variants of SARS-CoV-2 during the fifth wave in Pakistan

**DOI:** 10.3389/fpubh.2022.987452

**Published:** 2022-09-29

**Authors:** Zunera Jamal, Murtaza Haider, Aamer Ikram, Muhammad Salman, Muhammad Suleman Rana, Zaira Rehman, Syed Adnan Haider, Muhammad Ammar, Nadia Nisar, Massab Umair

**Affiliations:** Department of Virology, National Institute of Health, Islamabad, Pakistan

**Keywords:** vaccine breakthrough cases, Omicron and Delta variant, COVID-19 Pakistan, coronavirus in Pakistan, fifth wave in Pakistan

## Abstract

COVID-19 pandemic has severely affected Pakistan with 1,557,134 cases as of August 4, 2022. However, the data regarding breakthrough infections in Pakistan is scant. Hence, the objective was to analyze SARS-CoV-2 breakthrough infections with respect to vaccines and variants during the fifth wave in Pakistan. Therefore, the Department of Virology (NIH, Pakistan) genotyped 2,467 randomly selected individuals between November 2021 and February 2022 using the SNPsig^®^ SARS-CoV-2 (EscapePLEX) kit (PrimerDesign, UK). P681R and K417N mutations were used to distinguish delta and omicron. Data on the patient's age, gender, date of collection, variant, and vaccination status were analyzed using Statistical Package for Social Sciences (SPSS) software. Among 2,467 genotyped samples, Omicron was detected in 58.6% (*n* = 1445), Delta in 40.4% (*n* = 998) and undetermined/wildtype variant in 24 samples. The vaccination status of omicron-positive patients showed (49.7%; *n* = 718/1445) and Delta-positive patients (39.67%; *n* = 396/998) to be fully vaccinated. Of note, a high percentage 85% of breakthrough cases (*n* = 947) were identified among fully vaccinated individuals (*n* = 1114). Among them, 85.9% (*n* = 617/718) belonged to omicron and 83.3% (*n* = 330/396) to delta. Moreover, 76.7% (*n* = 855) of vaccinated individuals (*n* = 1114) received Sinopharm (*n* = 432) and Sinovac (*n* = 423) vaccines. The majority of breakthrough subjects who contracted Omicron were vaccinated with Sinopharm (93.0%, *n* = 256) and delta with Cansino (100%, *n* = 44). Individuals vaccinated with Sinovac showed the most frequent breakthrough cases for both Omicron and Delta variant between the 4th and 6th months (*n* = 278) after primary vaccination as compared to the 7th to 9th months (*n* = 24) category. While in case of Sinopharm, maximum breakthrough cases occurred between 7th to 9th months (*n* = 234) as compared to the 4th to 6th months (*n* = 120) after primary vaccination. Omicron and Delta breakthrough cases in men (*n* = 364 and 193) are more frequently seen than women (*n* = 253 and 138) respectively and breakthrough majority cases (*n* = 392) occurred in individuals aged 18–33 years. Breakthrough cases limiting monitoring in Pakistan impose a substantial constraint on policymakers' ability to take timely effective decisions. Since the current study consists of only a 2,467-genotyped sample, comprehensive data should be analyzed.

## Introduction

The COVID-19 outbreak has posed a significant challenge, with escalating morbidity and mortality, which so far infected 548,719,924 individuals with 6,350,355 fatalities globally (1). In Pakistan, as of June 26, 2022, the virus has infected 1,533,482 individuals, resulting in 30,368 deaths across the five waves. Pakistan has faced the fifth wave of SARS-CoV-2 in January 2022 that was driven by Omicron, with over 8,000 cases per day at its peak in late January (2). As variants emerge in the population, monitoring becomes a tremendous challenge for pandemic management and prevention *via* vaccination if the variant has increased virulence or transmissibility (3). Further numerous response strategies increases, such as expediting major vaccine rollouts and boosting vaccine immunogenicity by increasing vaccination doses (4).

Pakistan has administrated 259.9 million vaccine doses, as of June 26, 2022. Among these, 136.2 million individuals are partially vaccinated, 124.8 million are fully vaccinated, whereas 16.3 million have received a booster dose. Among the vaccines administered (Astrazeneca, Cansino, Moderna, Pfizer, Sinopharm, Sinovac, and Sputnik V), Sinopharm and Sinovac are the most widely used in Pakistan (5). Despite 82% of the eligible population is fully vaccinated, breakthrough cases have been reported in Pakistan (6, 7). On the other hand, there is no tracking of breakthrough cases in the fifth wave yet. However, COVID-19 breakthrough infection surveillance is not only essential for containing future outbreaks, but also determining the efficacy of vaccines against variants, and reducing hospitalization and death rates. A study has shown that parameters like case fatality rates are ineffective in tracking pandemics unless breakthrough infections are monitored in vaccinated individuals (8), who are still prone to spreading the virus (9). Consequently, it is essential to monitor breakthrough infections to advance the understanding of occurrence which is useful in devising pandemic management strategies (10). Therefore, this current study focuses on reporting breakthrough infections of vaccines during the fifth wave caused by Omicron and Delta in Pakistan.

## Methodology

### Sampling and data collection

Between Nov 2021, and Feb 2022, as part of routine surveillance, the oropharyngeal swab specimens of suspected subjects were collected in Viral Transport Medium and stored at 4°C at the Department of Virology, National Institute of Health (NIH), Islamabad. During the time period between Nov 20, 2021, and Feb 02, 2022, NIH successfully genotyped 2,467 SARS-CoV-2 randomly selected samples were, as part of routine surveillance in Pakistan. The information about the demographic data was recorded using a sample collection form at the time of sample collection and submitted to Laboratory information management system (LIMS). The internal control, positive control and negative control were run alongside during sample processing as a measure of quality control. Ethical approval was obtained from Institutional review board of NIH and written informed consent was obtained from study participants.

### RNA extraction and real-time PCR

The MagMAX Viral/Pathogen Nucleic Acid Isolation kit (ThermoFisher Scientific, USA) and the KingFisher Flex instrument (ThermoFisher Scientific, USA) were used for RNA extraction. Clinical RT-PCR testing was performed using TaqPath™ COVID-19 RT-PCR kit (ThermoFisher Scientific, USA) that targets the three genes (ORF1ab, N, and S). The PCR Genotyping Kit, SNPsig^®^ SARS-CoV-2 (EscapePLEX) kit (PrimerDesign, UK) targets K417N, E484K, and P681R mutations and in our study, it was used for the discrimination of Delta and Omicron variants. Inclusion criteria: samples from between November 20, 2021 and February 02, 2022, with a Cycle Threshold (CT) value of ≤37.

### Data management and analysis

The data regarding the patient's age, gender, district, sample's date of collection, variant detected, and vaccination status was initially entered into a Microsoft Excel spreadsheet, where plots were created, and then analyzed using the SPSS version 27.0 statistical software. To summarize and show the data, descriptive measures such as proportions and percentages were used. The Pearson Chi-square (χ2) test was used to confirm the existence of a relationship between vaccine types and months' interval (transformed categorical variable) as well as vaccine and variant with gender and age group. Independent sample *t*-test was used to compare breakthrough days with variants and vaccines type. Additionally, ANOVA was used to compare mean difference between “Vaccines and Breakthrough Days”. The level of significance for this study was set at 0.05 with a 95% confidence interval. When p <0.05, the results of this study were considered statistically significant. Furthermore, the cases were termed breakthrough since subjects got SARS-CoV-2 after the 14 days of being fully vaccinated.

## Results

Among 2,467 genotyped samples, Omicron was detected in 58.6% (*n* = 1,445) of samples comprising 871 males and 574 females, Delta was detected in 40.4% (*n* = 998) comprising 570 males and 428 females, and 24 samples had wildtype/undetermined variant. The vaccination status of omicron positive-patients (*n* = 1,445) showed 718 (49.7%) to be fully vaccinated, 31 were partially vaccinated, and 105 were unvaccinated. Among fully vaccinated individuals, 38.3% (*n* = 275) received Sinopharm and 36.6% (*n* = 263) Sinovac, 14.4% (*n* = 104) Cansino, 3.2% (*n* = 23) Astrazeneca, 2.6% (*n* = 19) Moderna, 2.5% (*n* = 18) Sputnik V, and 2.2% (*n* = 16) received Pfizer. While the vaccination status of individuals infected with delta (*n* = 998) showed 39.67% (*n* = 396) to be fully vaccinated, 24 were partially vaccinated and 169 were unvaccinated. Among vaccinated individuals, 40.4% (*n* = 160) received Sinovac, 39.6% (*n* = 157) Sinopharm, 11.1% (*n* = 44) Cansino, 3.3% (*n* = 13) Sputnik V, 2.8% (*n* = 11) Astrazeneca, 1.5% (*n* = 6) Moderna, 1.2% (*n* = 5) have been vaccinated with Pfizer (Supplementary Table 1; Supplementary Figure 1). Of note, Sinovac and Sinopharm were administered to the maximum number of people (*n* = 855) in the study.

During the current study, a high percentage (85%; *n* = 947/1,114) of breakthrough cases among the fully-vaccinated patients were identified. Among them, (85.9%; *n* = 617/718) belong to the omicron, whereas (83.3%; *n* = 330/396) were of delta instances. Of note, omicron showed higher ratio of breakthrough cases as compared to delta. Moreover, omicron breakthrough cases per vaccine showed Sinopharm to have maximum breakthrough cases (93.0%; *n* = 256), followed by Cansino (87.9%; *n* = 91), Astrazeneca (86.9%; *n* = 20), Sinovac (80.9%; *n* = 213), Moderna (78.9%; *n* = 15), Sputnik V (77.0%; *n* = 14), and Pfizer (50%; *n* = 8). While on contrary, Delta's breakthrough cases per vaccine showed Cansino to have maximum breakthrough cases (44; *n* = 100%). As per the order, Astrazeneca has (90%, *n* = 10), Sinopharm (89.8%; *n* = 140), Sinovac (76.2%; *n* = 122), Sputnik V (69.2%; *n* = 9), Moderna (50%; *n* = 3), and Pfizer (40%; *n* = 2) (Supplementary Table 2). The higher breakthrough rate recorded in Pakistan can be elucidated, in part, by the months intervals, gender and age distribution of the infected patients.

**Figure 1:**
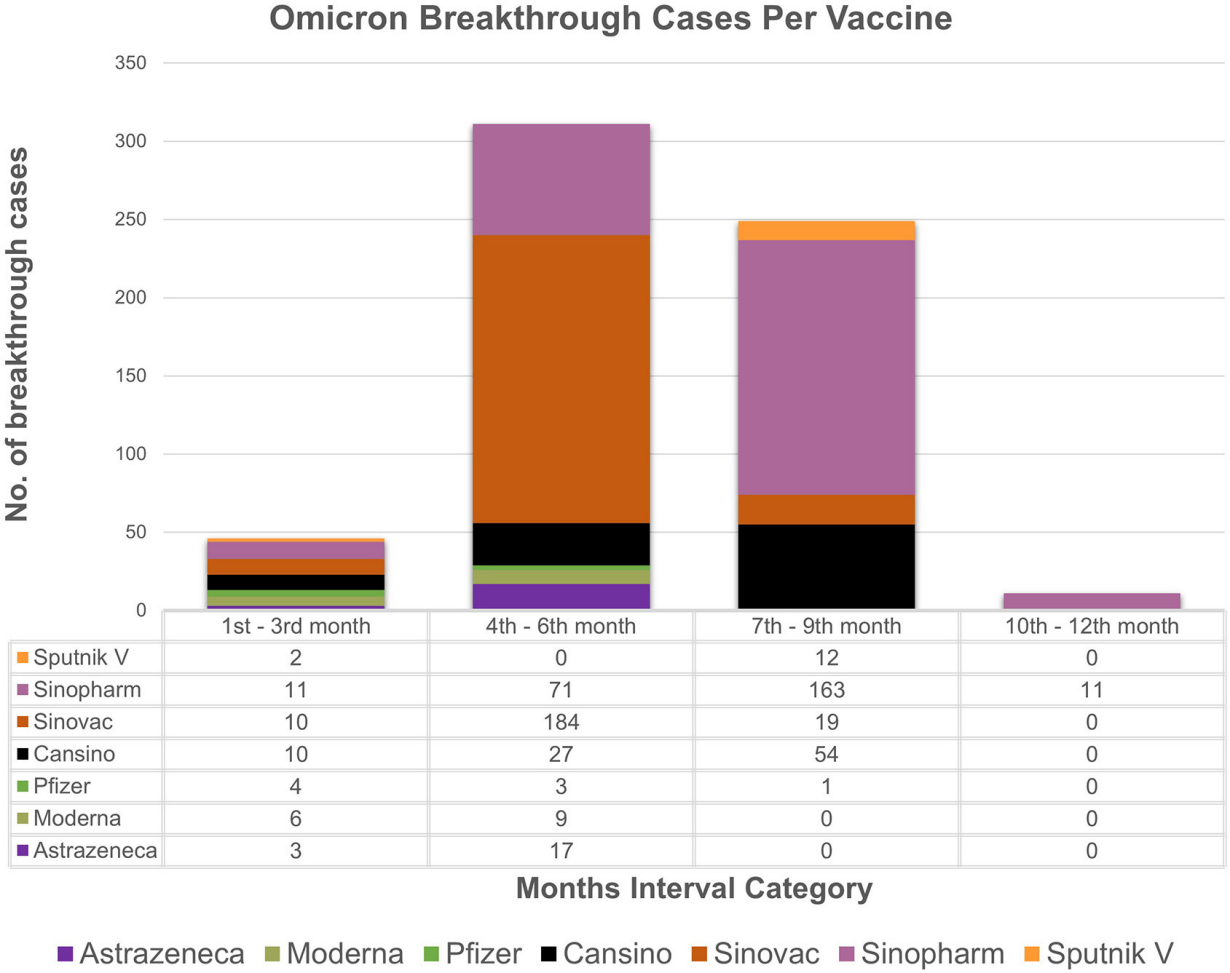
Breakthrough cases representation: the month's interval between being fully vaccinated and testing positive for omicron with respect to vaccines. X-axis represents month's interval while y-axis represents counts of breakthrough cases.

**Figure 2:**
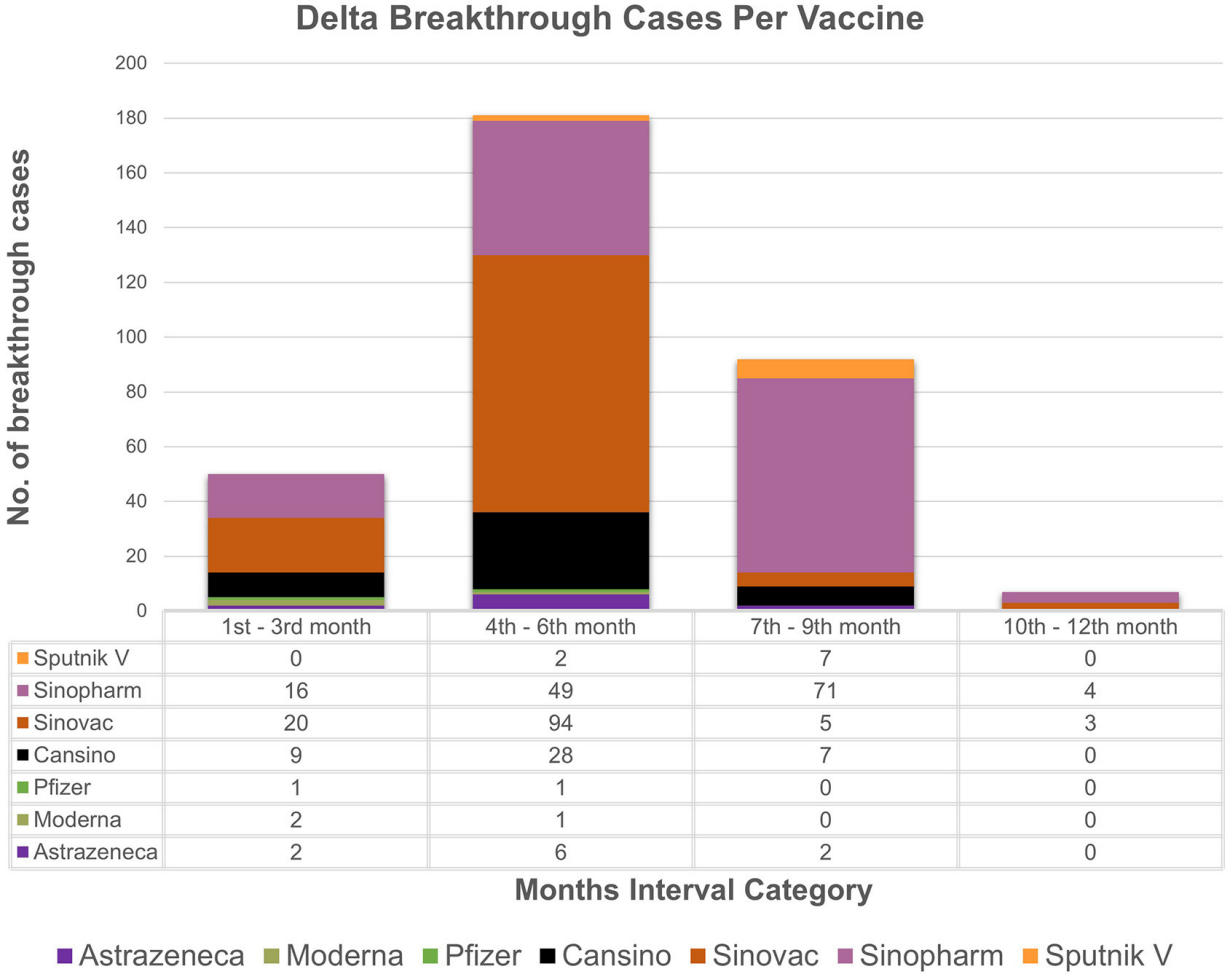
Breakthrough cases representation: the month's interval between being fully vaccinated and testing positive for delta with respect to vaccines. X-axis represents month's interval while y-axis represents counts of breakthrough cases.

The study analyzes the breakthrough data by examining the months' intervals between testing positive after being fully vaccinated. The chi-square association test between vaccine types and months' interval (transformed categorical variable) showed strong association (*p value* = *0.001*) between vaccine type and the months' interval. Based on the available data (Figures 1, 2), individuals vaccinated with Sinovac showed the most frequent breakthrough cases for both Omicron and Delta between the 4th and 6th months (*n* = 278) as compared to the 7th to 9th months (*n* = 24) category which is particular concern of this study. Contrarily, for Omicron or Delta-infected patients vaccinated with Sinopharm showed maximum number of breakthrough cases between the 7th and 9th months (*n* = 234) as compared to the 4th to 6th months (*n* = 120) category. The 76.7% (*n* = 855/1,114) of vaccinated individuals in the study received these two vaccines. Moreover, independent sample t-test to compare breakthrough days with variants and ANOVA comparing mean difference between “Vaccines and Breakthrough Days” showed corresponding *p-values 0.001* (Supplementary Tables 3, 4). Even so, research is needed to determine the effectiveness of the vaccine over a longer time.

**Figure 3:**
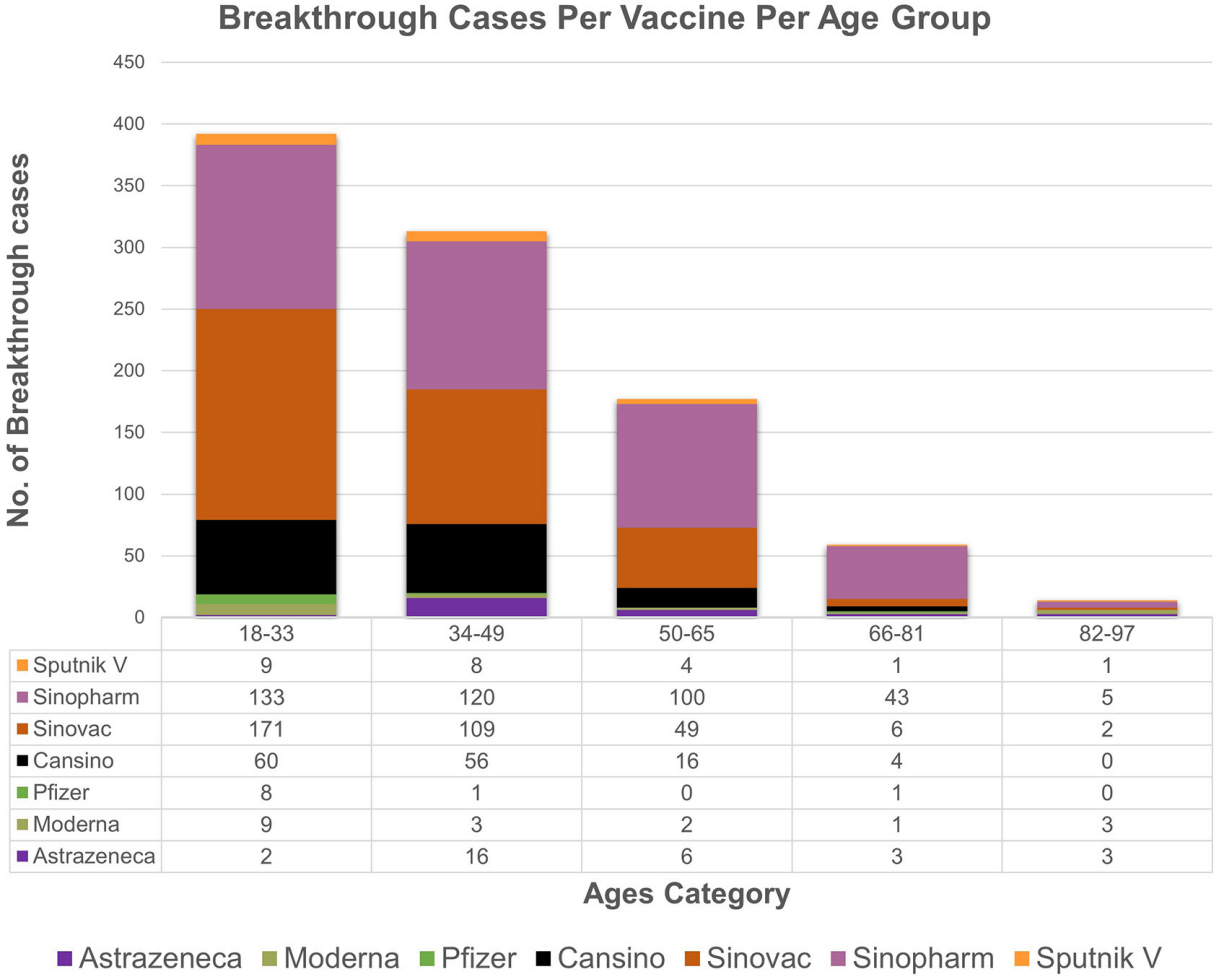
The age wise distribution of vaccines breakthrough cases. The majority of breakthrough cases occur in subjects aged 18–33(*n* = 392), followed by subjects aged 34–49 (*n* = 313).

The descriptive statistics of the gender-specific distribution of omicron with respect to vaccines among breakthrough cases show that cases in men (*n* = 364) are more frequently seen than women (*n* = 253). Likewise, delta cases were more frequent in men (*n* = 193) than in women (*n* = 138). However, the association between these variables (vaccine, gender) and (variant, gender) of breakthrough cases was not statistically significant as the corresponding *p-value (0.39,0.98)*, respectively, was greater than the level of significance (0.05). There is an association found between vaccine type and age groups as the observed *p value (0.001)* is less than level of significance (0.05). Moreover, age of included 956 patients ranged from 18 to 90 years across the study. The data (Figure 3) for all vaccines included in the study showed the overall majority (*n* = 392) of breakthrough cases occurred in subjects aged 18–33 years, followed by subjects (*n* = 313) aged 34–49. Notably, the most frequent cases (42 instances) were of 40-year-old patients.

As of February 22, 2022, the 20 of the 2,467 cases genotyped had contracted omicron and 27 had contracted delta, despite the administration of booster dose. Notably, the least difference between being omicron-positive following a booster shot is 5 days, and the maximum difference is 28 days, while the minimum difference for delta is 9 and the maximum is 62 days. Therefore, more data and continuous monitoring are indeed required to determine the duration of immunity following the booster and to understand difference of gene expression values between omicron and delta. The average CT values for Delta variant were 26.47 (ORF), 26.68 (N), 26.98 (S) and for Omicron were 24.57 (ORF), 24.68 (N), 25.54 (S). Furthermore, Spike gene was positive in 5.8% of Omicron and 94.2% of delta-positive genotyped samples.

## Discussion

Our 2,467 genotyped samples provides critical insights into vaccination status, breakthrough cases, and the age of individuals infected with strains circulating during the 5th wave in Pakistan. We highlight continued transmission of Variants of concern (VOCs) with highest number of Omicron cases followed by Delta variant between November 20, 2021 and February 02, 2022. This represents the highly transmissible nature of the omicron variant, supporting global observations that this rapidly spreading VOC is establishing itself as the dominant variant in countries such as the USA, UK and other countries (11–13). Despite being fully vaccinated, 49.7% of the individuals still got infected with Omicron and 39.67% with Delta. It is worth mentioning that the various studies proclaim vaccines to be highly effective at preventing serious illness. However, it is also evident that the antibody response elicited by these vaccines is transient, leading to breakthrough cases (14). Nevertheless, our study did not analyze/correlate disease severity with breakthrough infections.

Other study results have also suggested that Omicron breakthrough infections are less immunogenic than Delta, providing reduced protection against reinfection (15). As in line with the previous studies, our findings reveal that in comparison with delta variant infected patients' breakthrough cases (83.3%), Omicron-infected patients showed a slight higher number of breakthrough cases (85.9%). These increased vaccine breakthrough instances could be attributed to serologic and structural variations between Omicron and Delta (16). Furthermore, our study's descriptive data of omicron breakthrough cases per vaccine showed that Sinopharm had the most (93.0%; *n* = 256) and Pfizer had the fewest comparatively. However, overall Pfizer is administrated less frequently (*n* = 53,799,138) in Pakistan as compared to Sinopharm (*n* = 38,207,010) and Sinovac (*n* = 100,880,502), which are whole virus-based vaccines. Preliminary studies have shown that Pfizer or Moderna (mRNA-based vaccines) offer the most promising protection against both infection and hospitalization, in line with the recommendations of Center od Disease Control and Prevention(CDC) USA (17). It is crucial to note that more studies indicate that mRNA-based vaccines create the most antibodies; thus, breakthrough infections reported in individuals fully vaccinated with mRNA vaccines are lower than in individuals fully vaccinated with vector or whole genome-based vaccines (18). Another study, this time in Pakistan, on 2,868 Sinopharm-vaccinated people, found that the spike protein's average decrease rate was 1.71 AU/ml, or 1.66 BAU per day. Those who received the third dose showed a significant increase in spike protein concentrations exceeding 250 AU/ml. The 3–4 months following the second dosage (19). Several other studies have also indicated a drop in antibody response against other variants (20). Researchers also have found that Pfizer's two dosages gave 70% protection against hospitalization and 33% protection against omicron infection. This was a decrease from around 93% and 80% for the Delta variant, respectively (21). So far, one- or two-dose vaccines provide significantly less protection than those combined with a booster, as seen by increased rates of reinfections and breakthrough infections observed in several studies (22). As a result, breakthroughs are associated not only with vaccine type and the duration of immunity it provides, but also with variant (23). Therefore, the applicability of the stance “time-from-being fully vaccinated and incidence of breakthrough infection (months' intervals), the date of analysis” between Nov 20, 2021 and Feb 22, 2022 for omicron and delta variant was further tested.

Based on the analyzed breakthrough data, Sinovac showed the highest number of breakthrough cases for omicron (*n* = 184/617) and delta (*n* = 94/330) variant between the 4th and 6th month's category among the seven vaccines administrated in Pakistan as compared to 7th to 9th month category. Whereas, Sinopharm had the most breakthrough cases for omicron (*n* = 163/617) and delta (71/330) between the 7th and 9th month's category as compared to 4th and 6th months category. It is worth noting that just a few cases of breakthrough (*n* = 46/617, *n* = 50/330) occurred in the 1–3 months category for omicron and delta, respectively. This result ties well with a prior study wherein Khoury et al. demonstrated that antibody titers rose up to 24-fold after 1 month of the second dose of vaccine compared to day 1 of the second dose (24). The study strongly indicates a boost in immunity between 1 and 3 months after being fully vaccinated. However, it was also determined that antibody titers steadily decrease after 3 months, which indicates a drop in immunity. Interestingly, immunity between 1 to 3 months after full vaccination is lower in those over the age of 50, particularly in male participants. A similar pattern of results was obtained in several studies, indicating an elevated antibody titer in the first month followed by a rapid decline between 1 and 6 months (25, 26). Conclusively, these findings imply robust immunity approximately for a month, followed by a steady reduction in immunity. Therefore, based upon the analysis, it can be speculated that two doses of Sinopharm or Sinovac may not be sufficient to establish long-term protection. Furthermore, if a third dose of Sinopharm or a booster shot specifically Pfizer is given, antibody levels may improve significantly (27–29). While, in our study, as of February 22, 2022, only 84 individuals in our research had received booster doses. Despite receiving booster shots of Pfizer or Moderna, the least difference between being omicron-positive following a shot was 5 days, while for delta it was 9 days. In contrast to our findings and a report from Singapore, another study reported that no individual was infected after a booster (30, 31). Nonetheless, data is scarce on the efficacy of booster shots and the least number of days between becoming COVID positive even after shots. These findings support the notion that breakthrough infections are caused not only by constant mutations that result in immune escape and vaccine-induced antibody immune response, but also by other comorbidities (32, 33). Thus, this highlights the importance of developing better second-generation vaccines that may not necessitate frequent booster immunizations.

As of June 30, 2022, National Command and Operation Center (NCOC, Pakistan) recommendations state that the first booster dose should be administered 5 months after the last dose and the second booster dose 4 months later. While the CDC advises booster shots of either Pfizer-BioNTech or Moderna 3 months for those who are moderately or severely immunocompromised, they also advise getting a second booster injection 4 months after the first one (34). Additionally, the WHO's Strategic Advisory Group of Experts (SAGE) advises considering a booster dosage 4–6 months after the primary vaccination series is completed (35). Notably, just 26% of Pakistan's total eligible population and 3.4% of study population had received boosters. Given the high rate of fifth-wave breakthrough infections, booster doses should be increased, and the time between booster and primary vaccination should be reduced, as our data shows that the majority of breakthrough cases arose after 3 months of primary vaccination.

On the other hand, our data analysis of the significant association present between vaccine type and age of breakthrough cases showed Omicron's 45 and 33% of Delta patients belonging to the 18–33 age category, showing the younger being more infected. We speculate that the lower age is due to a disproportionately higher likelihood of delinquent behavior (e.g., less mask-wearing and less social distancing) in the younger population (16) as well as more exposure to outdoor activity (36). Contrarily, other studies manifest the age distribution of delta-infected patients with breakthrough infections to be skewed toward older age groups (37) with higher severity risk factors (38). Regardless, more research is needed to gain insights into the factors that contribute to the discrepancies between Delta and Omicron patients identified in this study. The current study showed similar finding that the average CT values for Delta were slightly higher than Omicron variant (39). However, investigations also indicated that there was no statistically significant difference in CT values based on lineages (40, 41).

Our study has several important limitations. First, as the Omicron and Delta variants were the dominant strains in Pakistan during the study period, the observed decrease in immunity duration provided by the vaccine against other strains cannot be deduced. Second, the number of cases is rapidly expanding, restricting our ability to investigate interactions between the factors under consideration. Third, the data assessed accounted for <1% of the Pakistani population, and participants under the age of 18 were excluded from the breakthrough data analysis; thus, the results may not be generalizable. Fourth, the vaccination status of omicron-positive (40.8%; *n* = 591/1,445) and delta-positive (40.9%; *n* = 409/998) patients is not known. Finally, the results could be affected by differences in health behaviors between the groups (such as social distancing and mask-wearing), a potential confounder that was not assessed.

Taken together, this genotyped study suggests a possible relative decrease in the long-term protection of the vaccines against the Omicron and Delta variants. This finding should be investigated in future studies, such as comparisons to long-term protection against different strains, as well as prospective clinical trials to investigate the effect of a booster vaccine against breakthrough infection and to reduce dose gaps to 3 months instead of 6 months after being fully vaccinated. Finally, variant trends are expected to be impacted by the lockdown measures implemented, vaccination status of affected individuals, therefore necessitating the genomic sequencing for controlling infection timely. However, the country's financial resources restrict it. Therefore, a robust response generated through genotype data analysis at the field level to implement quarantine and control measures is required.

## Data availability statement

The original contributions presented in the study are included in the article/Supplementary material, further inquiries can be directed to the corresponding author/s.

## Ethics statement

The studies involving human participants were reviewed and approved by Institutional Review Board of National Institute of Health, Islamabad, Pakistan. The patients/participants provided their written informed consent to participate in this study.

## Author contributions

AI and MU: conceptualization and resources. ZJ, MA, SH, and MU: methodology. ZJ, NN, and MU: formal analysis. ZJ and MU: writing original draft preparation. ZJ, MU, MH, MS, MR, ZR, MA, and SH: writing review and editing. All authors contributed to the article and approved the submitted version.

## Conflict of interest

The authors declare that the research was conducted in the absence of any commercial or financial relationships that could be construed as a potential conflict of interest.

## Publisher's note

All claims expressed in this article are solely those of the authors and do not necessarily represent those of their affiliated organizations, or those of the publisher, the editors and the reviewers. Any product that may be evaluated in this article, or claim that may be made by its manufacturer, is not guaranteed or endorsed by the publisher.
