# Supplementary material for: Breakthrough cases of Omicron and Delta variants of SARS-CoV-2 during the fifth wave in Pakistan
Source: Front Public Health. 2022 Sep 29;10:987452. doi: 10.3389/fpubh.2022.987452 (PMC9557048; doi:10.3389/fpubh.2022.987452)
Supplement: Supplementary file 1 [file Data_Sheet_1.docx]

Supplementary Table 1: Genotyped samples (n=2467) of SARS-CoV-2 variants (Omicron, Delta) during fifth wave (November 2021 and February 2022) in Pakistan

| **Count Of Gender Per Variant** | | | | | | |
| --- | --- | --- | --- | --- | --- | --- |
| Gender | Omicron | | Delta | | Wild Type/ Undetermined | |
| Female | 574 | | 428 | | 11 | |
| Male | 871 | | 570 | | 13 | |
| Grand Total | 1445 | | 998 | | 24 | |
|  | | | | | | |
| **Vaccination Status Per Variant** | | | | | | |
| Status | Omicron | | Delta | | Wild Type/ Undetermined | |
|  | Count | Percentage | Count | Percentage | Count | Percentage |
| Fully vaccinated | 718 | 49.7% | 396 | 39.7% | 12 | 50% |
| Partially vaccinated | 31 | 2.14% | 24 | 2.40% | 0 | 0% |
| Not vaccinated | 105 | 7.27% | 169 | 16.9% | 4 | 16.6% |
| Undetermined | 591 | 40.8% | 409 | 40.98% | 8 | 33.3% |
| Grand Total(count) | 1445 | | 998 | | 24 | |
|  | | | | | | |
| **Vaccines Administrated To Fully Vaccinated Individuals Per Variant** | | | | | | |
| Vaccines | Omicron | | Delta | | Wild Type/ Undetermined | |
|  | Count | Percentage | Count | Percentage | Count | Percentage |
| Sinopharm | 275 | 38.3% | 157 | 39.6% | 6 | 25% |
| Sinovac | 263 | 36.6% | 160 | 40.4% | 4 | 16.66% |
| Cansino | 104 | 14.4% | 44 | 3.3% | 1 | 4.16% |
| Astrazeneca | 23 | 3.2% | 11 | 2.8% | 0 | 0% |
| Moderna | 19 | 2.6% | 6 | 1.5% | 0 | 0% |
| Sputnik V | 18 | 2.5% | 13 | 3.3% | 0 | 0% |
| Pfizer | 16 | 2.2% | 5 | 1.2% | 1 | 4.16% |
| GrandTotal_count | 718 |  | 396 |  | 24 |  |

Supplementary Table 2 Breakthrough infections (n=947) per variant and vaccine of genotyped samples during the fifth wave in Pakistan

| **Breakthrough Infections Per Variant** | | | | | | | |
| --- | --- | --- | --- | --- | --- | --- | --- |
|  | Omicron | | | Delta | | | Total |
| Breakthrough cases | 617 | | | 330 | | | 947 |
| Vaccinated individuals | 718 | | | 396 | | | 1114 |
| Percentage | 85.9% | | | 83.3% | | | 85% |
| **Breakthrough Infections Per Vaccine Per Variant** | | | | | | | |
| Vaccines | Omicron | | | Delta | | | |
|  | Breakthrough Count | Vaccine Count | Percentage | Breakthrough  Count | | Vaccine Count | Percentage |
| Sinopharm | 256 | 275 | 93.0% | 140 | 157 | | 89.8% |
| Sinovac | 213 | 263 | 80.9% | 122 | 160 | | 76.2% |
| Cansino | 91 | 104 | 87.9% | 44 | 44 | | 100% |
| Astrazeneca | 20 | 23 | 86.9% | 10 | 11 | | 90% |
| Moderna | 15 | 19 | 78.9% | 3 | 6 | | 50% |
| Sputnik V | 14 | 18 | 77.4% | 9 | 13 | | 69.2% |
| Pfizer | 8 | 16 | 50% | 2 | 5 | | 40% |
| Total | 617 | 718 | 85.9% | 330 | 396 | | 83.3% |

Supplementary Table 3 Statistical breakthrough data analysis (Independent sample t test) between variants and days of being Omicron/Delta-positive after being fully vaccinated.

| Variant | Number of Samples | Range (Days) | Mean ± SD (Days) | *p*-value |
| --- | --- | --- | --- | --- |
| Delta | 330 | 27-366 | 168 ± 61.36 | *0.001* |
| Omicron | 617 | 16-313 | 187 ± 61.46 |  |

Supplementary Table 4 Statistical breakthrough data analysis (ANOVA) between vaccines and days of being Omicron/Delta-positive after being fully vaccinated.

| Vaccines | Number of Samples | Mean ± SD (Days) | Range (Days) | *p*-value |
| --- | --- | --- | --- | --- |
| **Astrazeneca** | 30 | 134 ± 37 | 56-231 | *0.001* |
| **Cansino** | 135 | 175 ± 59 | 16-286 |  |
| **Moderna** | 18 | 92 ± 37 | 34-135 |  |
| **Pfizer** | 10 | 113 ± 77 | 28-281 |  |
| **Sinopharm** | 396 | 210 ± 63 | 34-313 |  |
| **Sinovac** | 335 | 157 ± 42 | 27-366 |  |
| **Sputnik V** | 23 | 210 ± 67 | 20-261 |  |

*
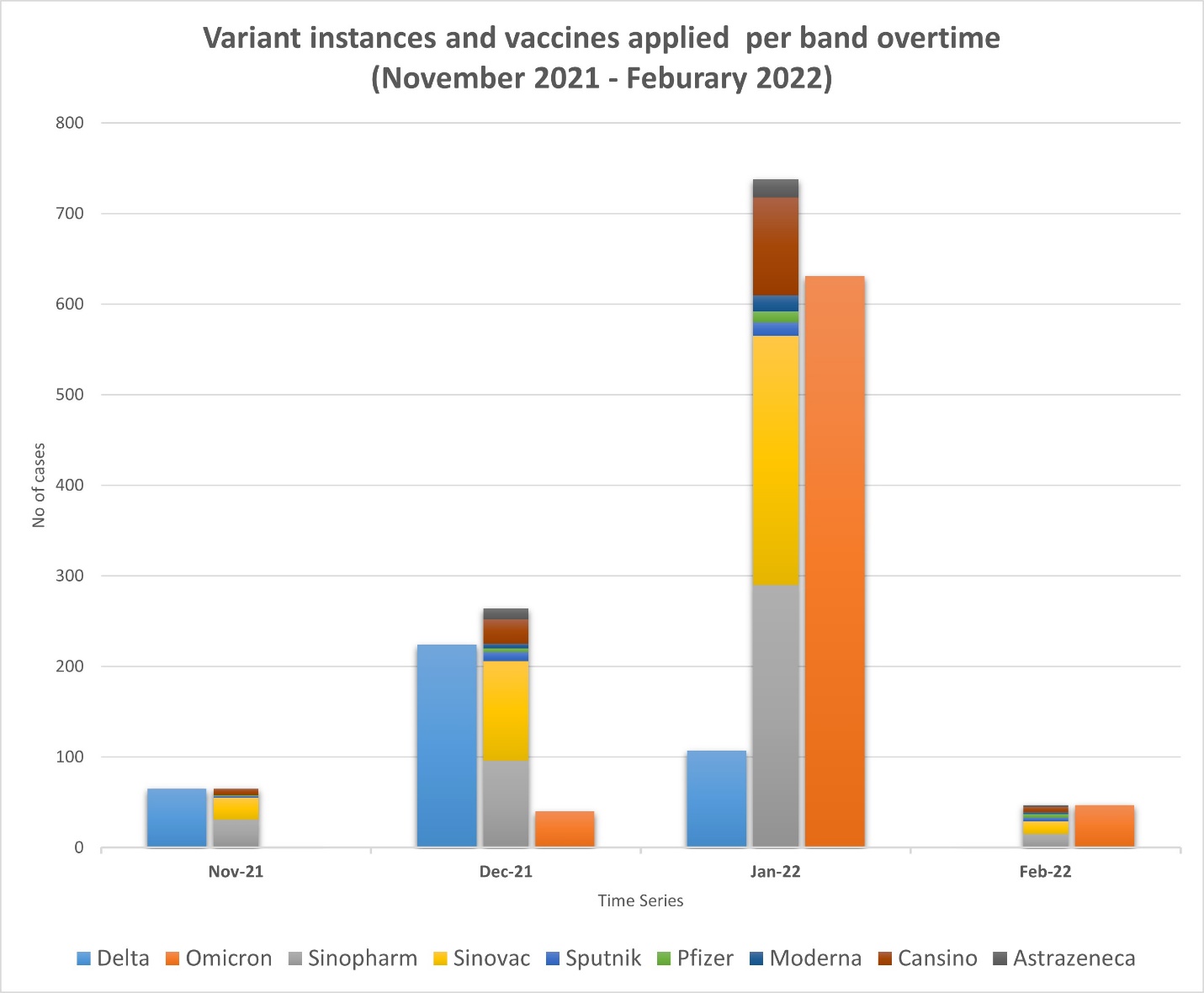
Supplementary Figure 1: No of cases of fully-vaccinated individuals infected with Omicron and Delta and vaccines applied per brand to them over time (Nov 2021 – Feb 2022)*
